# Evaluating the comfort of robot paths in varying-width corridors: passenger vs. observer perspectives

**DOI:** 10.3389/frobt.2026.1971439

**Published:** 2026-10-29

**Authors:** Fumiya Ohnishi, Gloria Beraldo, Masaki Takahashi

**Affiliations:** 1 School of Science for Open and Environmental Systems, Graduate School of Science and Technology, Keio University, Yokohama, Japan; 2 Institute of Cognitive Sciences and Technologies, National Research Council of Italy (CNR-ISTC), Rome, Italy

**Keywords:** corridor navigation, human comfort, human-robot spatial interaction, human–robot spatial interaction, path planning, personal mobility robot

## Abstract

In corridors shared by humans and robots, mobile robots should follow paths that are comfortable for passengers and observers. Previous studies have examined straight corridors with uniform width; however, actual environments include varying-width corridors, such as hallways connected to open spaces or containing static obstacles. Comfortable path design in such environments is unclear, and differences between passenger and observer preferences remain underexplored. This study provides initial, context-specific empirical evidence on comfort preferences among robot paths in varying-width corridors from passenger and observer perspectives. Through experiments (n = 35), three paths were examined: a straight wall-following path, a center path with a small heading change, and a wall-following path with a larger heading change. Each path was evaluated in two perspectives (passenger and observer) and two environments with sudden or gradual width changes. Comfort was measured by ranking the three paths. A Plackett–Luce model was used to test within-condition path preferences, the effects of perspective and environment, and their interaction. Across all conditions, the straight wall-following path was mainly preferred. In the sudden-change environment, path preference differed significantly between perspectives: passengers preferred the center path over the path with the larger heading change, whereas observers showed no such preference and rated the latter relatively higher. No significant effect of environment or interaction was detected. These findings suggest that, within the scenarios tested, smaller heading changes are associated with comfort, whereas less center-aligned motion may be particularly relevant when observer comfort is prioritized.

## 1 Introduction

In recent years, autonomous mobile robots such as delivery robots and personal mobility devices have been increasingly deployed in human-shared environments (Singamaneni et al., 2024). To integrate these robots into daily use, their motion should be comfortable for both onboard passengers and nearby observers who perceive the robot’s motion from a third-person perspective. In this paper, comfort is defined, in line with the definition provided by previous work (Kruse et al., 2013; Francis et al., 2025), as the *absence of annoyance and stress for humans in interactions with robots*. In autonomous navigation, a robot typically first plans a global path to its goal on a map and then computes velocity commands to follow that path while considering its current position and surrounding obstacles. Consequently, global path design is crucial in determining how the robot moves and, by extension, how humans evaluate its motion. Thus, developing global paths that prioritize the comfort of both passengers and nearby observers is an important challenge in modern robotics.

Most prior studies on comfort-oriented robot path design in corridor environments have focused on straight corridors with uniform width (Morales et al., 2013; Robinson et al., 2024). However, real indoor spaces often contain width changes caused by open spaces, irregular wall geometry, or static obstacles. Examples of such varying-width corridors are presented in Figure 1. In such settings, the visible spatial structure changes dynamically as the robot moves, potentially leading passengers and observers to interpret the robot’s motion differently than they would in straight, uniform-width corridors.

**FIGURE 1:**
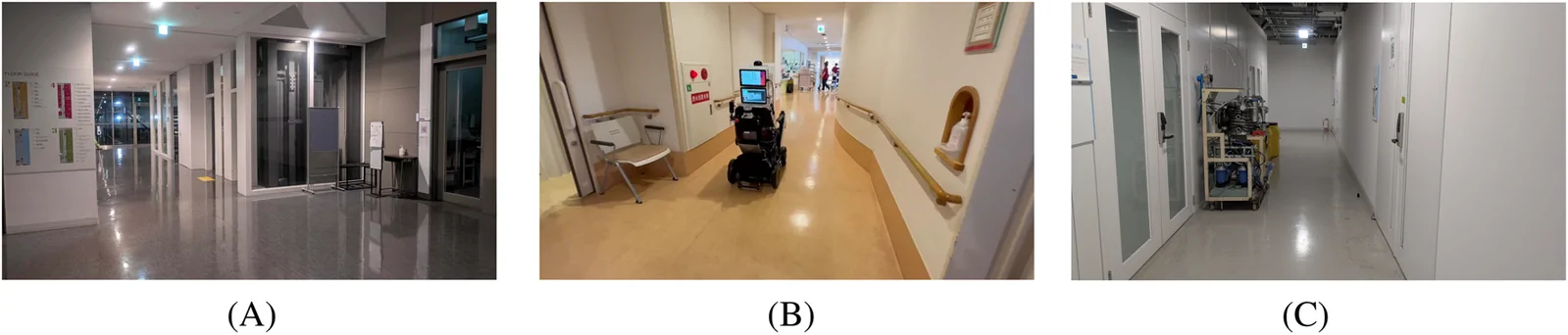
Examples of varying-width corridors in real settings. **(A)** Hallway connected to open space. **(B)** Corridor with non-uniform walls. **(C)** Corridor with static obstacles.

This raises two unresolved questions. First, path-design principles for uniform corridors—such as avoiding the centerline (Morales et al., 2013) or following a wall (Robinson et al., 2024)—may be unsuitable for varying-width corridors. In non-uniform layouts, centerline paths may maximize geometric clearance, but they may also appear unnecessarily central or ambiguous. Wall-following introduces an additional design choice: whether to follow a wall with changing geometry or a wall that remains straight. Moreover, the more comfortable option may differ between gradual width changes, which allow continuous wall following, and sudden width changes, which interrupt it. Figure 2 illustrates this ambiguity. Second, prior studies have typically evaluated comfort from either the passenger perspective or the observer perspective, with few direct comparisons under matched conditions. Thus, comparing the two perspectives under the same conditions is important for assessing whether a common path planner can be aligned with both passengers and observers or whether different use cases may require different planning strategies.

**FIGURE 2:**
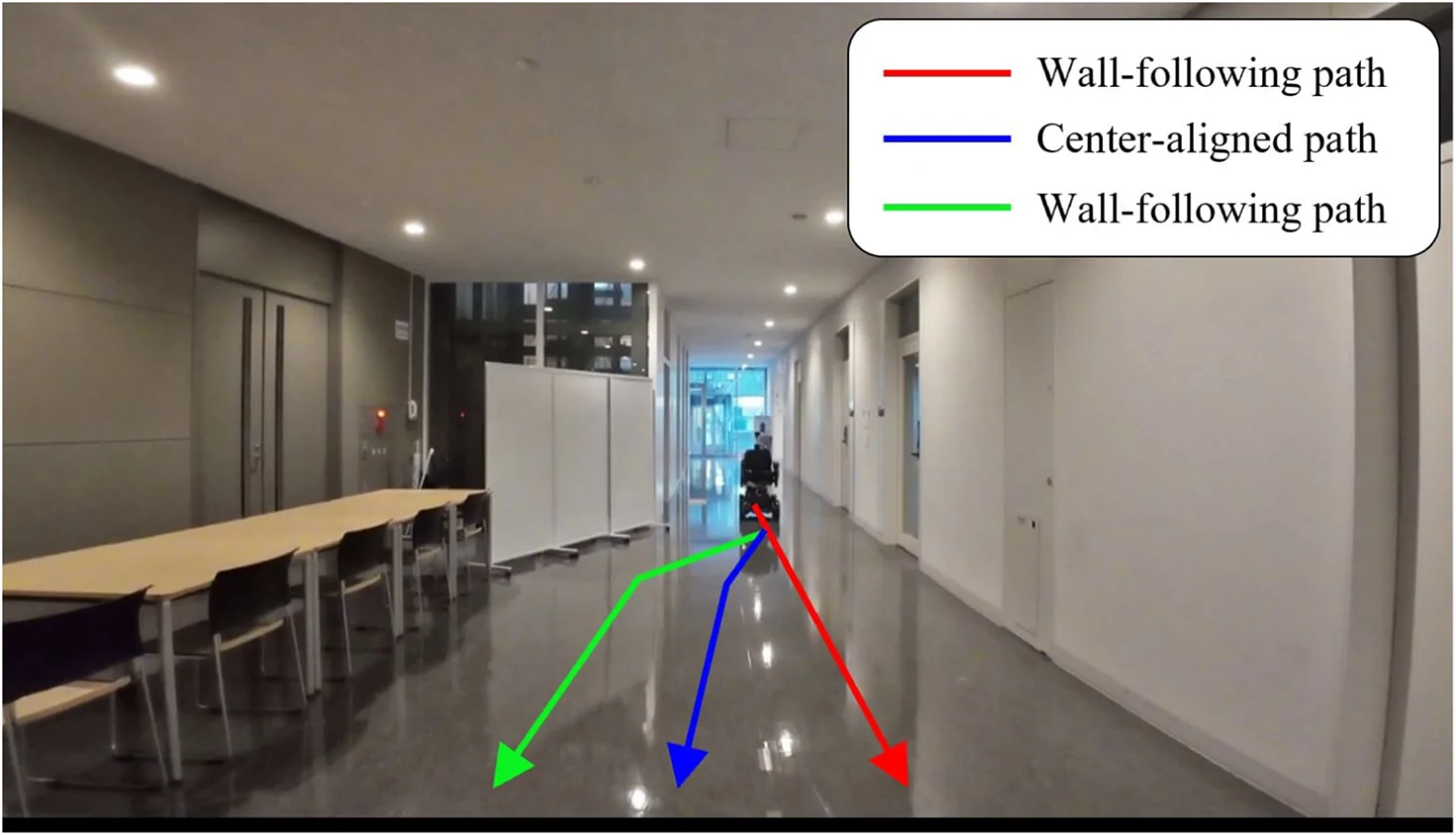
Candidate paths in a varying-width corridor. Previous studies suggest that, in corridors with uniform width, wall-following paths that avoid the center tend to be more comfortable, but the effectiveness of this strategy in varying-width environments remains unclear. This figure illustrates a center-aligned path (blue) and two wall-following paths (red and green) defined by different target walls, highlighting the ambiguity in wall selection.

The objective of this study is to identify comfortable navigation paths in varying-width corridors and to determine how their evaluation changes with perspective and environment. This study provides initial, context-specific empirical evidence on comfort preferences among robot paths in varying-width corridors from passenger and observer perspectives.

In summary, this paper addresses the following research questions.

- RQ1: Which tested path is preferred within each Environment × Perspective condition?
- RQ2: Do path preferences differ between passenger and observer perspectives?
- RQ3: Do path preferences differ between the two corridor layouts?
- RQ4: Does the perspective effect depend on the corridor layout?

The remainder of this paper is structured as follows. Section 2 reviews related work. Section 3 describes the experimental setup and methods. Section 4 explains the data analysis. Section 5 reports the results. Section 6 discusses the findings with respect to RQ1–RQ4. Section 7 summarizes the limitations. Finally, Section 8 concludes the paper.

## 2 Related work

This study lies within the field of socially aware (or human-aware) robot navigation, which aims to generate motion that is not only physically safe but also acceptable to people who share the environment with the robot (Kruse et al., 2013; Singamaneni et al., 2024; Francis et al., 2025). Previous work includes proxemics-based approaches that account for personal space and comfortable passing distances (Pacchierotti et al., 2006; Neggers et al., 2022a; b; Nikolovska et al., 2026) and approaches based on the social force model that predict pedestrian motion and plan navigation accordingly (Helbing and Molnár, 1995; Ferrer et al., 2013). Learning-based approaches include reinforcement-learning methods for socially compliant navigation in crowds (Chen et al., 2017; 2019), vision-based policies trained on human–robot interaction data (Hirose et al., 2024), and, more recently, methods based on large language models and vision–language models that use scene understanding and language-based reasoning to generate socially acceptable robot motion (Song et al., 2025; Payandeh et al., 2025). However, many of these studies focused primarily on local avoidance or short-horizon motion generation in encounters with nearby people, whereas the present study focuses on global path planning itself—namely, where in the environment the robot should travel.

Several studies have examined how robot motion affects comfort in indoor navigation. Prior work evaluated robot passing distance and speed (Pacchierotti et al., 2006; Neggers et al., 2022b; a), pedestrian avoidance (Ohnishi and Takahashi, 2024; Neggers et al., 2024), and path design in corridors (Morales et al., 2013; 2017; 2018; Robinson et al., 2024; Greenberg et al., 2025). For passengers, Morales et al. (2013) showed that comfort in straight corridors depends on the wheelchair’s lateral position. Morales et al. (2017) combined a passenger comfort model with a pedestrian walking model to plan wheelchair paths in straight corridors, and both passengers and passing pedestrians rated the resulting side-positioned path higher than a shortest path. Morales et al. (2018) further demonstrated that reduced visibility in occluded spaces can increase passenger discomfort. For observers, Robinson et al. (2024) reported that wall-following paths were rated more favorably than shortest-path motion in straight corridors, and Greenberg et al. (2025) found that path curvature affects affective responses in hallway navigation. In contrast, corridor layouts with varying width remain relatively underexplored.

Previous studies have examined the social acceptance of robot motion from either the passenger’s or the observer’s perspective. Research on the passenger perspective has addressed wheelchair path planning oriented to passenger comfort (Morales et al., 2013; 2018) or to both passenger and pedestrian comfort (Morales et al., 2017), comfortable pedestrian-avoidance motion for personal mobility vehicles (Yoshitake et al., 2023), side-by-side wheelchair navigation with caregivers (Nguyen et al., 2018), and quantitative comfort criteria based on acceleration and jerk (De Winkel et al., 2023). Studies on the observer perspective have examined how third parties interpret robot motion (Robinson et al., 2024; Greenberg et al., 2025; Zhang et al., 2019; Neggers et al., 2022a; Gupta et al., 2024), including how perceived comfort depends on observer position and the robot’s pedestrian-avoidance motion (Ohnishi and Takahashi, 2024). Observer-aware planning has also aimed to improve visibility and legibility by keeping the robot within the observer’s field of view (Taylor et al., 2022) or by reducing ambiguity regarding its intended destination (Amirian et al., 2024). However, to the best of our knowledge, no prior study has directly compared how the same robot motion is perceived by passengers and stationary observers under matched conditions, even though such comparisons are important for designing path planners that balance both perspectives.

Furthermore, it is useful to relate comfort-oriented path design to conventional global planning strategies. Clearance-maximizing planners based on inflated obstacle costs (Lu et al., 2014) or Voronoi diagrams (Garrido et al., 2006) tend to favor center-aligned motion. Additionally, wall-following has a long history of use as a navigation heuristic and has recently been revisited from the viewpoint of social acceptance (McGuire et al., 2019; Robinson et al., 2024). Representative formulations include Bug-family methods (McGuire et al., 2019) and the Extended Voronoi Graph, which can generate wall-proximal paths (Beeson et al., 2005). The present study builds upon these lines of work by testing how center-oriented and wall-oriented paths are perceived in corridors with variable widths along the path and by comparing passenger and observer evaluations within the same experimental framework.

## 3 Experimental setup and methods

### 3.1 Scenario and environments

This study focuses on a scenario in which a passenger utilizes an autonomous wheelchair in a human-shared environment, such as an airport or hospital. The passenger is assumed to select a destination area (e.g., a boarding gate at an airport) through a user interface. Consequently, the passenger knows the direction of travel but not the detailed position of the destination (e.g., its exact coordinates) or the path the wheelchair will take.

In contrast, observers are bystanders who view the wheelchair from an external, third-person perspective. They can see the direction of travel and the corridor geometry, but they do not know the detailed position of the destination or the path the wheelchair will follow.

Figure 3 illustrates the experimental layouts, including the two corridor environments, the three candidate paths, and the observer position (green circular marker). Two environments were examined: Environment A, which features a sudden change in corridor width, and Environment B, which features gradual changes in width. Photographs of the scenes are provided in the Supplementary Figures S1, S2.

**FIGURE 3:**
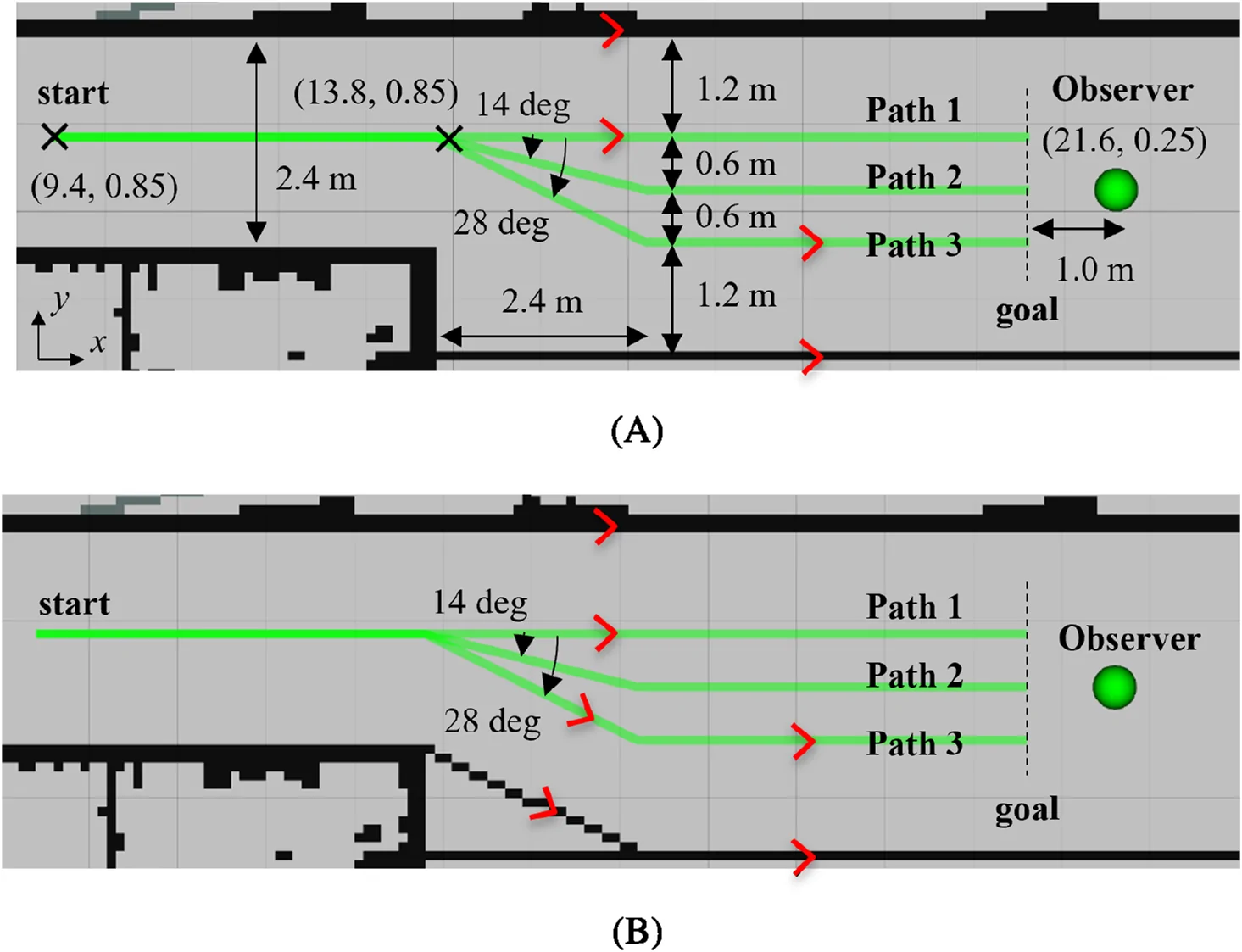
Experimental settings investigated in this study. Environment A features a sudden change in corridor width, whereas Environment B presents a gradual change. The robot passenger’s paths are plotted in green. Path 1 follows a straight wall and involves no heading change. Path 2 runs along the center of the corridor. Path 3 follows a wall and includes a heading change; in Environment B, this path consistently stays close to the wall. The observer is positioned at the center of the corridor after the width change (i.e., green circular marker). **(A)** Environment A. **(B)** Environment B.

### 3.2 Robot and candidate paths

Three path types were evaluated. Path 1: Wall-following with no wall shape variation and no heading change. Path 2: A central path with a 14° heading change. Path 3: Wall-following with wall shape variation and a 28° heading change. These representative paths were selected to compare practically relevant path types in varying-width corridors rather than to isolate the causal contributions of individual geometric features. The heading-change angles for Paths 2 and 3 were chosen so that the forward distance traveled during the turning motion matched the original corridor width. After the heading change, Path 3 was aligned to maintain the same wall distance as that before the corridor widened, whereas Path 2 was configured to pass through the corridor center. Path 3 also differed across environments in terms of how it followed the wall. In Environment A, although the path ultimately follows the wall after entering the widened corridor, it does not maintain wall-following behavior during the heading change. In Environment B, by contrast, the path follows the wall throughout.

All paths started from the same location to prevent participants from forming expectations based on the starting point. The observer’s position and orientation were set at the center of the corridor after the width change, facing the region where the corridor geometry changed. This configuration ensured that the corridor appeared symmetric from the observer’s viewpoint. Global paths were provided to the robot in advance. Path tracking was performed using the pure pursuit algorithm (Coulter, 1992). The robot was a WHILL Model CR configured to travel at a constant speed of 0.5 m/s and 1.0 rad/s with accelerations of 0.5 m/s^2^ and 1.5 rad/s^2^, respectively. The dimensions of the robot were 985 mm (length) × 550 mm (width) × 740–940 mm (height).

Videos of all conditions are provided in the Supplementary Table S1.

### 3.3 Participants and experimental design

This study consisted of an in-person survey (n=35) designed to compare comfort rankings of the three candidate paths from both passenger and observer perspectives across the two corridor layouts. The experiment was approved by the Bioethics Committee of the Faculty of Science and Technology, Keio University (Approval Nos. 2024-053 and 2025-017). All participants provided written informed consent. All methods were performed in accordance with the ethical principles of the Declaration of Helsinki and relevant institutional guidelines and regulations of Keio University.

Thirty-five participants (28 males and 7 females, $M_{\text{age}}=23.2$ , $SD_{\text{age}}=1.9$ , age range: 20–30) participated in the in-person survey. All participants were Japanese. Participants were recruited through an open call distributed to students enrolled at Keio University, and interested students voluntarily registered through an online recruitment form; participation was not compulsory. Their reported frequencies of encountering mobile robots were as follows: 13 at least once a week, 13 once a week to once a month, 5 once a month to once every 6 months, and 4 once every 6 months to once a year.

The within-subjects design crossed the two environments, the two perspectives, and the three paths (Sections 3.1 and 3.2). Each participant completed all 12 conditions, yielding 420 in-person navigation trials in total. The order of the four environment–perspective blocks and the order of the three paths within each block were randomized across participants. In passenger blocks, participants rode the robot directly. In observer blocks, they watched the robot from the designated location shown in Figure 3.

Before the experiment, participants received detailed instructions consistent with the scenario in Section 3.1. Participants were informed that each individual would perform the experiment twice: once as a passenger and once as an observer. As a passenger, the participant sat in the wheelchair, and as an observer, the participant watched the movement of the wheelchair. In both roles, the written instructions stated that the participant knew the direction of travel but not the detailed position of the destination, which corresponds to the scenario of Section 3.1. For both roles, participants were required to evaluate their preferences along the path based on perceived comfort, defined as the absence of annoyance and stress for humans in interaction with robots. In the passenger role, participants were instructed not to evaluate ride quality (i.e., platform-specific characteristics such as vehicle-specific vibration and the sensation of speed). In both roles, they were instructed not to evaluate the robot’s speed, to treat doors in the experimental environment as walls, and to disregard possible pedestrians emerging from doors.

### 3.4 Procedure

Participants were informed about the study procedure and provided written informed consent. They then completed a short demographic questionnaire covering age, gender, and familiarity with mobile robots.

Within each environment–perspective block, participants experienced the robot’s motion along the three paths in a randomized order and then ranked the three paths in terms of comfort. To avoid imposing a fixed response-entry order or encouraging careless responses, the display order of the response fields in the questionnaire was also randomized. Participants assigned unique ranks from 1 to 3, with no duplicate ranks allowed. After completing the evaluations, participants were invited to provide optional free-text comments.

The total time required to experience the three paths within one environment–perspective block was approximately 3 min and 40 s. The total duration of the experiment was approximately 20 min per participant.

### 3.5 Measures

The dependent variable was perceived comfort. For each combination of environment and perspective, participants ranked the three paths (Path 1, Path 2, and Path 3) in terms of perceived comfort. Because the objective was to develop design guidelines for more comfortable paths, a ranking approach was used to elicit clearer relative preferences among the three alternatives.

Participants were also invited to answer three optional free-text questions about perspective-related differences, environment-related differences, and the comfort level of each path.

The independent variables were Path (Path 1, Path 2, and Path 3), Perspective (Passenger vs. Observer), and Environment (Sudden width change (Environment A) vs. Gradual width change (Environment B)). The questionnaire used in the in-person experiment (Japanese version) is available online at https://forms.gle/jf4jV6TWHM7tNLAJ8.

## 4 Data analysis

The analysis used complete rankings of the three paths (Path 1, Path 2, and Path 3) for each combination of Perspective (Passenger vs. Observer) and Environment (A vs. B), in which every participant ranked the paths from most to least comfortable with no ties. Because each analyzed response was a full ordering of all three alternatives, reducing the data to first-place counts alone or to separately analyzed pairwise choices would neglect information. Therefore, for the inferential analysis, a Plackett–Luce (PL) ranking model, which is designed for ranking data, was used (Luce, 1959; Plackett, 1975): all odds ratios, confidence intervals, and tests reported in Section 5 derive from this model (Sections 4.1 and Section 4.2). In addition, for the descriptive analysis, Borda scores (Section 4.3) are used as visual summaries of the rankings.

### 4.1 Statistical model

The PL model treats a ranking as a sequence of choices from shrinking choice sets. Let $\pi =\left(\pi_{1},\pi_{2},\pi_{3}\right)$ denote an observed ranking, where $\pi_{1}$ is the top-ranked path and $\pi_{3}$ is the bottom-ranked path. The probability of that ranking is given by Equation 1:

$$\mathrm{Pr}\left(\pi \right)=\frac{\exp\left(U_{\pi_{1}}\right)}{\exp\left(U_{\pi_{1}}\right)+\exp\left(U_{\pi_{2}}\right)+\exp\left(U_{\pi_{3}}\right)}\cdot \frac{\exp\left(U_{\pi_{2}}\right)}{\exp\left(U_{\pi_{2}}\right)+\exp\left(U_{\pi_{3}}\right)},$$ (1)

because the first stage selects one path from all three candidates, the second stage selects one path from the two remaining candidates, and the final stage has a probability of 1 because only one path remains. Here, $U_{k}$ is the latent utility (or relative worth) of path k . A larger $U_{k}$ indicates that the path is more likely to be chosen earlier in the ranking.

For identifiability, Path 1 was used as the reference alternative $\left(U_{1}=0\right)$ . Let E∈{0,1} denote Environment (EnvA =0 , EnvB =1 ), and let P∈{0,1} denote Perspective (Passenger =0 , Observer =1 ). The utilities for Path 2 and Path 3 were modeled as in Equations 2–4:

$$U_{1}=0,$$ (2)

$$U_{2}\left(E,P\right)=\beta_{2}+\beta_{2E}E+\beta_{2P}P+\beta_{\mathrm{2EP}}EP,$$ (3)

$$U_{3}\left(E,P\right)=\beta_{3}+\beta_{3E}E+\beta_{3P}P+\beta_{\mathrm{3EP}}EP.$$ (4)

Under this parameterization, $\beta_{2}$ and $\beta_{3}$ represent the baseline log-odds that Path 2 or Path 3 is chosen ahead of Path 1 in the reference condition (EnvA–Passenger). The coefficients with subscript E represent how those log-odds change in EnvB for passengers, those with subscript P represent how they change for observers in EnvA, and those with subscript EP represent the additional observer-by-environment interaction. For non-reference conditions, the relevant log-odds are obtained by adding the corresponding terms; for example, in EnvB–Observer, the Path 2 vs. Path 3 log-odds are $\left(\beta_{2}+\beta_{2E}+\beta_{2P}+\beta_{\mathrm{2EP}}\right)-\left(\beta_{3}+\beta_{3E}+\beta_{3P}+\beta_{\mathrm{3EP}}\right)$ .

Let L denote the log-odds contrast for a given comparison, where a contrast is a pre-specified linear combination of coefficients (e.g., Path 2 vs. Path 3 in EnvB–Observer, as illustrated above). The corresponding odds ratio is OR=exp(L) . Thus, OR =1 indicates equal odds, OR >1 indicates that the first path in the contrast is more likely to be chosen ahead of the second, and OR <1 indicates the reverse.

Parameters were estimated using maximum likelihood.

### 4.2 Statistical hypothesis testing

Four pre-specified questions were tested: (i) path differences within each Environment × Perspective condition (RQ1); (ii) perspective effects within each environment (RQ2); (iii) environment effects within each perspective (RQ3); and (iv) the three-way interaction among path, perspective, and environment (RQ4).

Each question was formulated as a linear hypothesis on the estimated coefficient vector β , namely $H_{0}:A\beta =0$ , where A is a contrast matrix.

Omnibus tests were conducted using Wald block tests. The $\chi^{2}$ statistics reported in Table 1 were computed as in Equation 5:

$$W={\left(A\hat{\beta }\right)}^{⊤}{\left[A\;\hat{Var}\left(\hat{\beta }\right)\;A^{⊤}\right]}^{-1}\left(A\hat{\beta }\right),$$ (5)

where $\hat{\beta }$ is the estimated coefficient vector of the fitted PL model, and $\hat{Var}\left(\hat{\beta }\right)$ denotes its participant-clustered sandwich covariance estimator. Under $H_{0}$ , W is asymptotically $\chi^{2}$ distributed with degrees of freedom equal to rank(A) . In this study, all omnibus tests had 2 degrees of freedom. With three paths and Path 1 set as the reference, only two path contrasts are linearly independent (Path 2 vs. Path 1 and Path 3 vs. Path 1), whereas the remaining contrast (Path 2 vs. Path 3) is their difference. Therefore, although three pairwise path comparisons can be reported *post hoc*, each omnibus test is based on only two independent quantities.

**TABLE 1:** Omnibus Wald block tests based on the participant-clustered sandwich covariance estimator.

Effect	Condition	$\chi^{2}$	p	What the test asks
Path differences	EnvA passenger	27.00	<0.001 ***	Whether comfort differs by path (at least one pair) in Environment A among passengers
Path differences	EnvA observer	19.13	<0.001 ***	Whether comfort differs by path (at least one pair) in Environment A among observers
Path differences	EnvB passenger	15.25	<0.001 ***	Whether comfort differs by path (at least one pair) in Environment B among passengers
Path differences	EnvB observer	9.65	0.008**	Whether comfort differs by path (at least one pair) in Environment B among observers
Perspective effect (Obs vs. pas)	EnvA	10.47	0.005**	Whether perspective (observer vs. passenger) changes comfort for at least one path pair in Environment A
Perspective effect (Obs vs. pas)	EnvB	2.90	0.235	Whether perspective (observer vs. passenger) changes comfort for at least one path pair in Environment B
Environment effect (B vs. A)	Passenger	4.40	0.111	Whether environment (B vs. A) changes comfort for at least one path pair among passengers
Environment effect (B vs. A)	Observer	3.60	0.165	Whether environment (B vs. A) changes comfort for at least one path pair among observers
Three-way interaction $\left(\Delta \Delta_{p}\right)$	All	4.83	0.089	Whether the observer–passenger comfort difference changes between environments for at least one path pair

Each $\chi^{2}$ value is a 2-df Wald statistic for the corresponding two-contrast null hypothesis. A significant result indicates that at least one of the two jointly tested contrasts differs from zero. Significance codes: * p<0.05 , ** p<0.01 , *** p<0.001 .

In a significant omnibus test, at least one of the jointly tested independent contrasts differs from zero, but the specific contrast is not identified. When an omnibus test was significant, *post hoc* pairwise contrasts were examined using Wald tests, and Holm correction was applied within each *post hoc* family to control the family-wise error rate (Holm, 1979).

Because each participant contributed multiple rankings across conditions, observations by a participant were not independent. Therefore, a participant-clustered sandwich covariance estimator was used in all Wald tests, following sandwich covariance inference for likelihood-based estimators (White, 1982) and cluster-robust inference for grouped observations (Cameron and Miller, 2015).

### 4.3 Descriptive summaries

Mean Borda scores under the 3/2/1 rule summarize the observed rankings descriptively, and their model-predicted counterparts allow a visual check of the model fit. The computation of the model-predicted expected Borda scores is described below. The model-predicted first-place probability for Path i in condition (E,P) is

$$p_{i,1}\left(E,P\right)=\mathrm{Pr}\left(R_{i}=1|E,P\right)=\frac{w_{i}\left(E,P\right)}{W\left(E,P\right)}.$$

Here, $w_{i}\left(E,P\right)=\exp\left\{U_{i}\left(E,P\right)\right\}$ and $W\left(E,P\right)=w_{1}\left(E,P\right)+w_{2}\left(E,P\right)+w_{3}\left(E,P\right)$ . Let j and k denote the other two paths. The model-predicted second-place probability is

$$p_{i,2}\left(E,P\right)=\frac{w_{j}\left(E,P\right)}{W\left(E,P\right)}\frac{w_{i}\left(E,P\right)}{w_{i}\left(E,P\right)+w_{k}\left(E,P\right)}+\frac{w_{k}\left(E,P\right)}{W\left(E,P\right)}\frac{w_{i}\left(E,P\right)}{w_{i}\left(E,P\right)+w_{j}\left(E,P\right)},$$

and $p_{i,3}\left(E,P\right)=1-p_{i,1}\left(E,P\right)-p_{i,2}\left(E,P\right)$ . The expected Borda score under the 3/2/1 rule is

$$E\left[B_{i}|E,P\right]=3p_{i,1}\left(E,P\right)+2p_{i,2}\left(E,P\right)+p_{i,3}\left(E,P\right).$$

For observed data in condition (E,P) , let $n_{EP}$ be the number of valid rankings and let $n_{i,1},n_{i,2},n_{i,3}$ be the counts of Path i ranked 1st, 2nd, and 3rd. Then

$${\hat{B}}_{i}\left(E,P\right)=\frac{3n_{i,1}+2n_{i,2}+n_{i,3}}{n_{EP}}.$$

## 5 Results

### 5.1 Model fit and descriptive summaries

The in-person dataset comprised n=35 participants and 140 complete rankings. The fitted PL model provided a better fit than an equal-probability null model, as indicated by the higher log-likelihood ( ${\text{LL}}_{\text{full}}=-210.36$ vs. ${\text{LL}}_{\text{null}}=-250.85$ ). Table 2 presents the coefficient estimates.

**TABLE 2:** Coefficient estimates from the PL model. Reference condition: EnvA–Passenger, with Path 1 as the baseline alternative. SE denotes the participant-clustered sandwich standard error of the coefficient estimate. OR =exp(coef) .

Parameter	coef.	SE	OR [95% CI]
$\beta_{2}$	−1.206	0.357	0.30 [0.15, 0.60]
$\beta_{3}$	−2.083	0.406	0.13 [0.06, 0.28]
$\beta_{2E}$	−0.109	0.365	0.90 [0.44, 1.84]
$\beta_{3E}$	0.651	0.448	1.92 [0.80, 4.62]
$\beta_{2P}$	−0.240	0.419	0.79 [0.35, 1.79]
$\beta_{3P}$	0.878	0.368	2.41 [1.17, 4.95]
$\beta_{\mathrm{2EP}}$	0.645	0.416	1.91 [0.84, 4.31]
$\beta_{\mathrm{3EP}}$	−0.161	0.508	0.85 [0.32, 2.30]

Figure 4 summarizes the Borda scores under the 3/2/1 scoring rule, with bars denoting the mean observed Borda scores and points denoting the model-predicted expected Borda scores (Section 4.3). Figure 5 visualizes the mean observed Borda scores mapped onto the paths actually traversed by the robot in the experiment.

**FIGURE 4:**
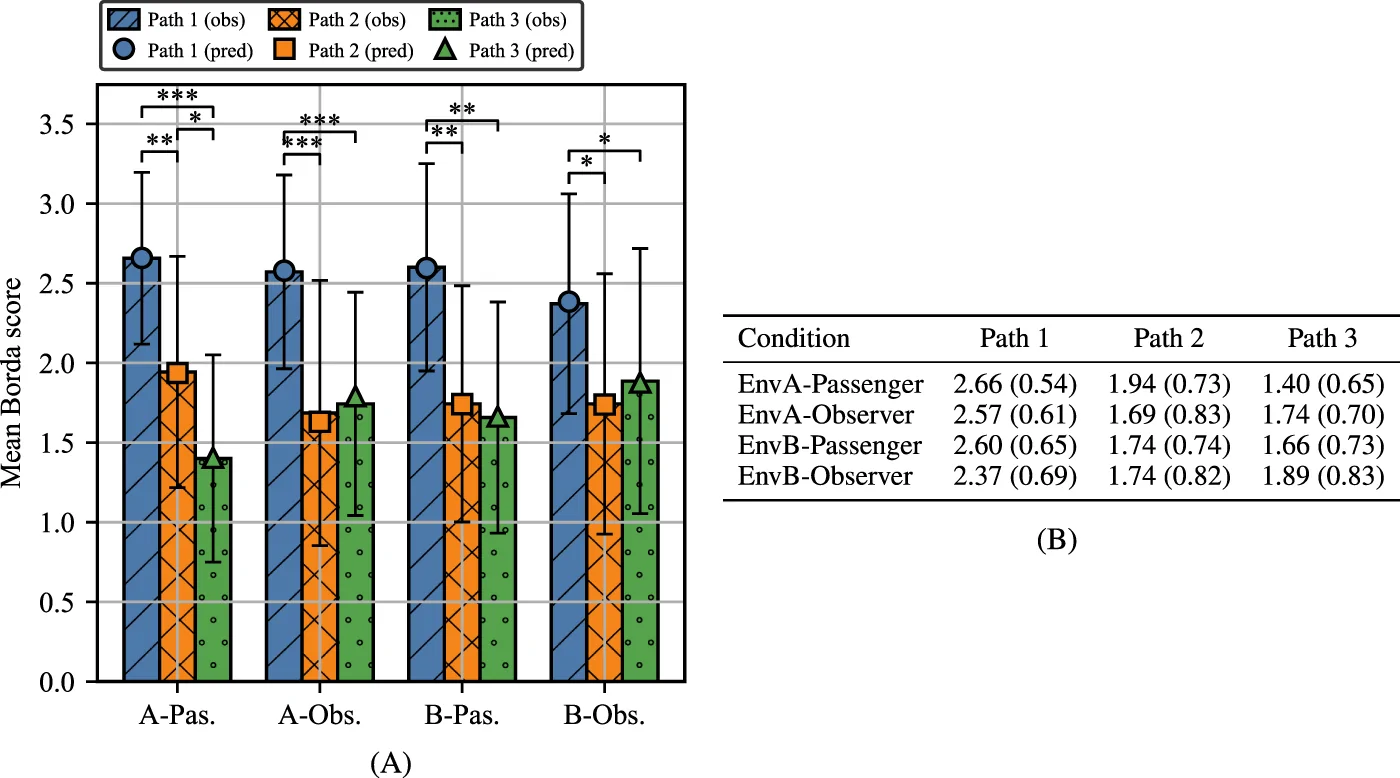
Borda-score summaries from the in-person experiment. The left panel visualizes the model-predicted and observed mean Borda scores, and the right panel reports the corresponding observed descriptive statistics. **(A)** Mean Borda scores (3/2/1) observed in the data (bars) and predicted by the fitted model (points), shown separately for each Environment × Perspective condition. Asterisks indicate pairs with significant differences in the *post hoc* tests described in Section 5.2 (* p<0.05 , ** p<0.01 , *** p<0.001 ). Error bars indicate ±1 SD. **(B)** Observed mean Borda scores (3/2/1) for each Environment × Perspective condition. Values indicate the mean (SD).

**FIGURE 5:**
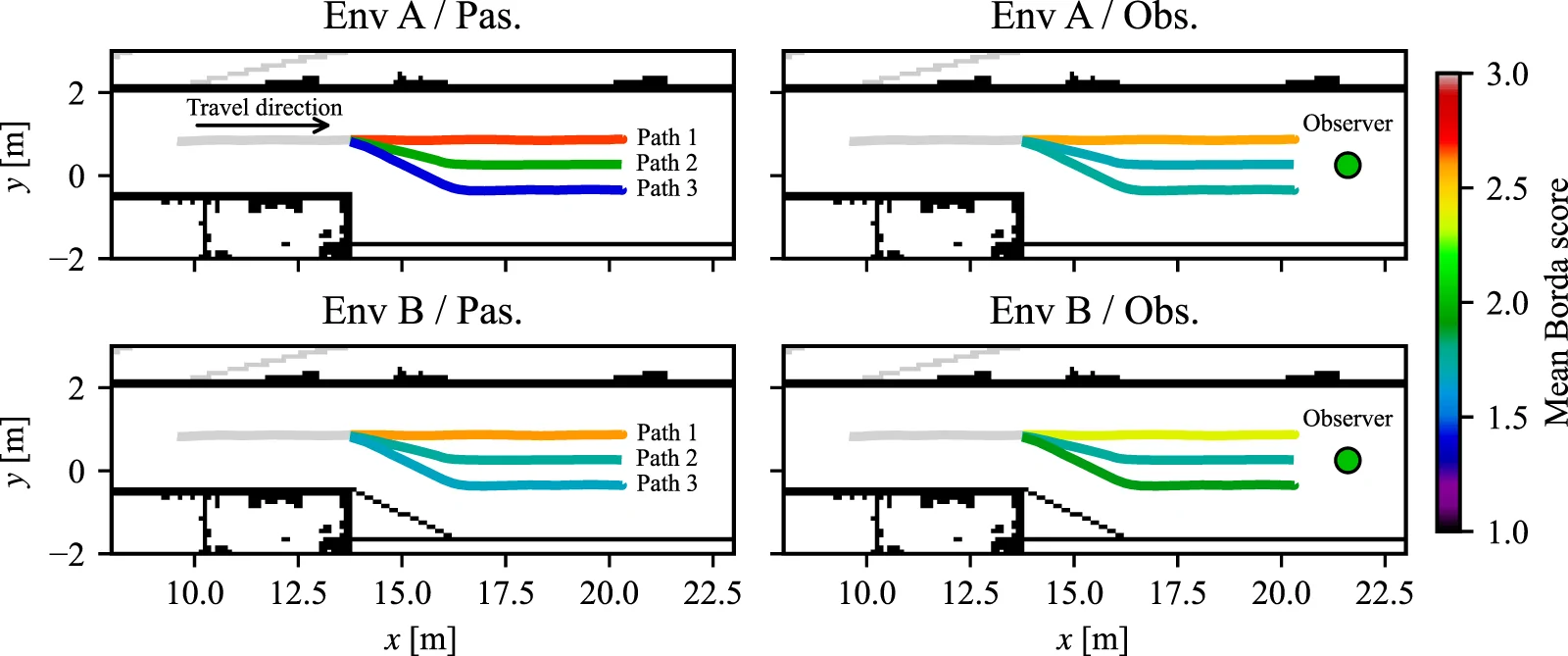
Comfort maps by environment and perspective, created by mapping participant-averaged mean observed Borda scores onto the paths actually traversed by the robot. Path 1 consistently received the highest scores, and Path 3 was rated higher by observers than by passengers. The green circle in the observer panels marks the observer position.

Figures 4, 5 show that Path 1 was consistently preferred across all conditions. By contrast, the relative ordering of Paths 2 and 3 varied by perspective—passengers tended to rank Path 2 above Path 3, whereas observers tended to rank Path 3 above Path 2—and this pattern was only partially supported inferentially.

### 5.2 Path differences within each environment-by-perspective condition (RQ1)

Within each Environment × Perspective condition, the first omnibus test asked whether Path 2 and Path 3 jointly differed from the Path 1 baseline. The omnibus null hypothesis was as given in Equation 6:

$$H_{0}:\;\log OR\left(\text{Path}2\text{ vs Path}1\right)=0\;\wedge \;\log OR\left(\text{Path}3\text{ vs Path}1\right)=0.$$ (6)

A significant result indicates that at least one of those two condition-specific path contrasts differs from zero.

Descriptive summaries in Figure 4 show that Path 1 received the highest mean score in every condition. Omnibus tests confirmed path differences in all four conditions (all $\chi^{2}\left(2\right)\geq 9.65$ , all p≤0.008 ; Table 1). Post hoc contrasts in Figure 6 showed a consistent pattern across conditions: Path 1 was preferred over both Path 2 and Path 3. The Path 2 vs. Path 3 contrast was condition-dependent. Path 2 was only preferred over Path 3 in EnvA–Passenger (OR =2.40 , 95% CI [1.15, 5.04], Holm-adjusted p=0.020 ), whereas the remaining three conditions showed no supported Path 2–Path 3 difference.

**FIGURE 6:**
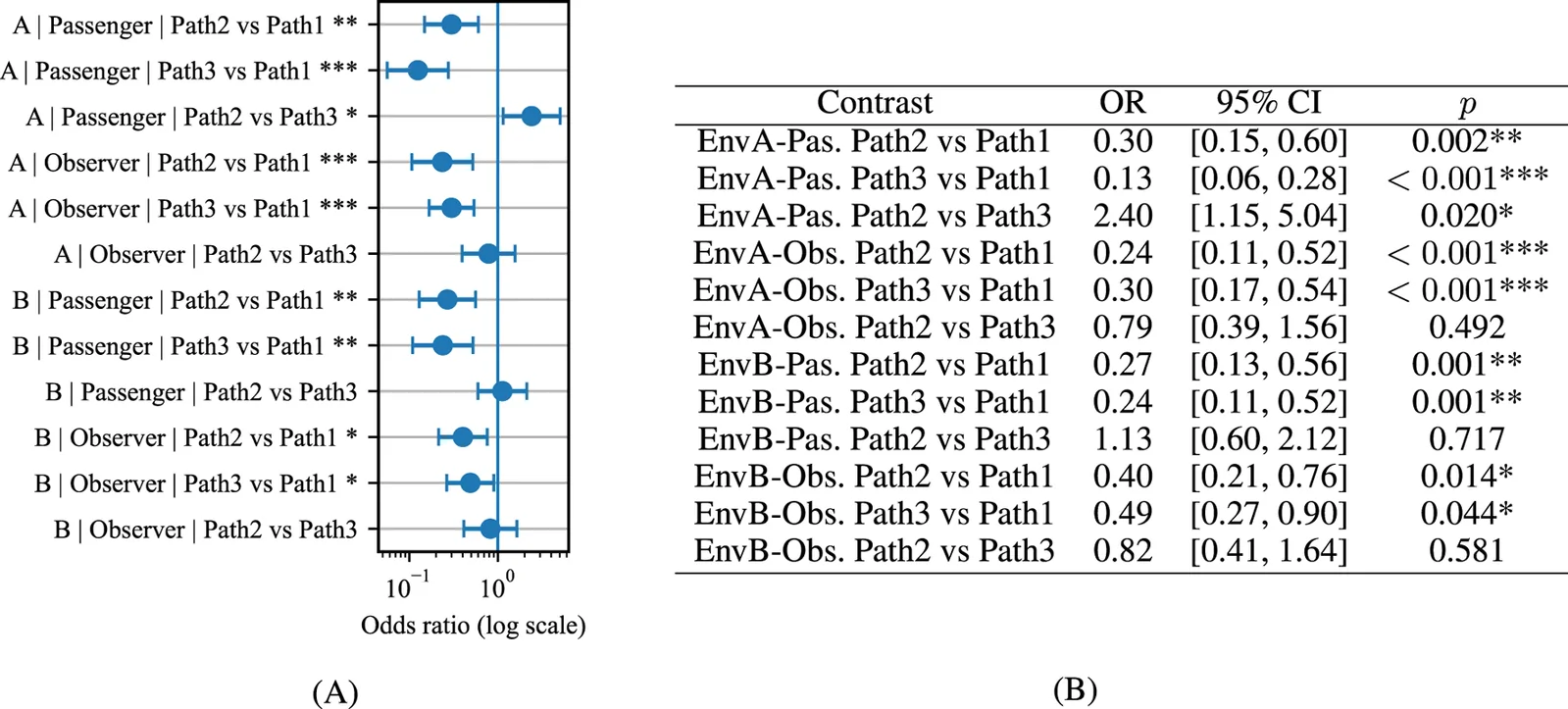
Within-condition path contrasts for each Environment × Perspective condition. The left panel presents the forest plot as odds ratios (log scale) with 95% confidence intervals based on participant-clustered sandwich standard errors. The right panel shows the corresponding *post hoc* statistics (odds ratios, 95% confidence intervals, and Holm-adjusted p values). Values below 1 indicate lower relative preference for the first path in the contrast. **(A)** Forest plot. **(B)** Post hoc statistics.

In summary, Path 1 was significantly preferred over Paths 2 and 3 in all conditions, and a clear Path 2 > Path 3 difference appeared only for passengers in Environment A.

### 5.3 Perspective effects within each environment (RQ2)

For RQ2, the omnibus test asked whether path preferences differed between observers and passengers within each environment. For each environment, the omnibus null hypothesis was as given in Equation 7:

$$H_{0}:\;\Delta_{p}\left(\text{Path}2\text{ vs Path}1\right)=0\;\wedge \;\Delta_{p}\left(\text{Path}3\text{ vs Path}1\right)=0,$$ (7)

where $\Delta_{p}$ denotes Observer minus Passenger on the log-odds scale.

Regarding the preference order within each perspective, descriptive results in Figure 4 exhibit a consistent ordering across environments: in both environments, passengers ranked the paths as Path 1 > Path 2 > Path 3, whereas observers ranked them as Path 1 > Path 3 > Path 2. This pattern indicates that the relative ordering of Paths 2 and 3 was reversed between perspectives. Omnibus tests revealed an overall perspective effect in EnvA ( $\chi^{2}\left(2\right)=10.47$ , p=0.005 ; Table 1) but not in EnvB ( $\chi^{2}\left(2\right)=2.90$ , p=0.235 ). Within EnvA, *post hoc* contrasts in Figure 7 showed that, relative to Path 1, observers favored Path 3 more than passengers did (OR =2.41 , 95% CI [1.17, 4.95], Holm-adjusted p=0.034 ). The same *post hoc* analysis also indicated a lower relative preference for Path 2 vs. Path 3 among observers than among passengers (OR =0.33 , 95% CI [0.16, 0.69], Holm-adjusted p=0.010 ).

**FIGURE 7:**
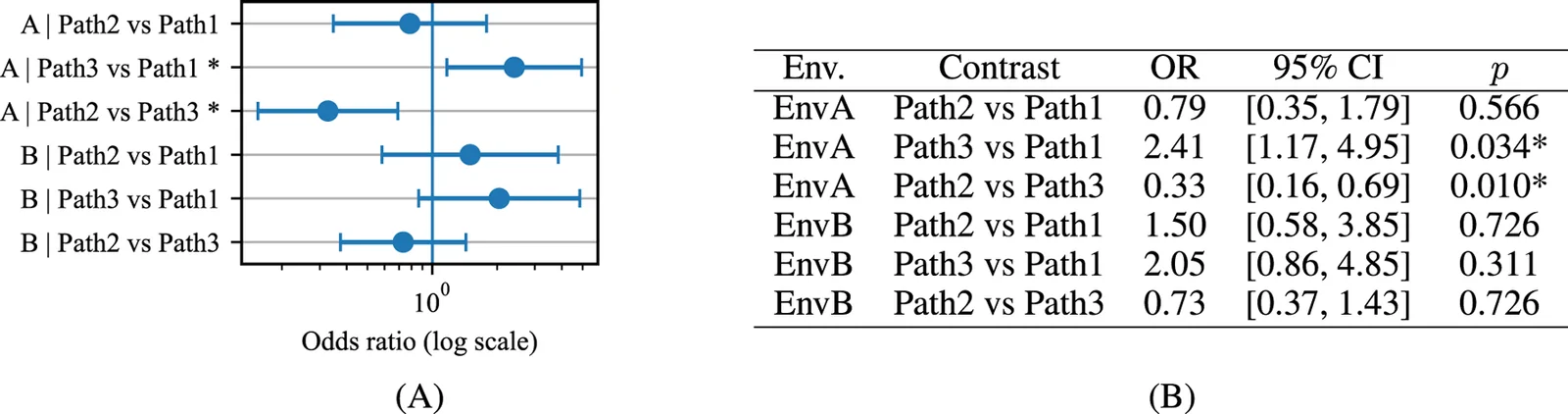
Perspective effects within each environment (Observer vs. Passenger). The left panel shows the forest plot as odds ratios (log scale) with 95% confidence intervals based on participant-clustered sandwich standard errors. The right panel presents the corresponding *post hoc* statistics (odds ratios, 95% confidence intervals, and Holm-adjusted p values). **(A)** Forest plot. **(B)** Post hoc statistics.

In summary, in the sudden-width-change environment (EnvA), the perspective effect on path comfort was significant. In terms of the relative shift between perspectives, observers showed a significantly stronger preference for Path 3 over Path 1 and a significantly weaker preference for Path 2 over Path 3 than passengers did.

### 5.4 Environment effects within each perspective (RQ3)

For RQ3, the omnibus test asked whether path preferences differed between EnvA and EnvB within each perspective. The omnibus null hypothesis was as given in Equation 8:

$$H_{0}:\;\Delta_{e}\left(\text{Path}2\text{ vs Path}1\right)=0\;\wedge \;\Delta_{e}\left(\text{Path}3\text{ vs Path}1\right)=0,$$ (8)

where $\Delta_{e}$ denotes EnvB minus EnvA on the log-odds scale.

Descriptive results from Figure 4 indicate no substantial environment-dependent change in the preference ordering. Omnibus tests showed no overall environment effect for either perspective (Passenger: $\chi^{2}\left(2\right)=4.40$ , p=0.111 ; Observer: $\chi^{2}\left(2\right)=3.60$ , p=0.165 ; Table 1). Thus, Figure 8 (panels A and B) provides descriptive OR estimates rather than confirmed environment effects.

**FIGURE 8:**
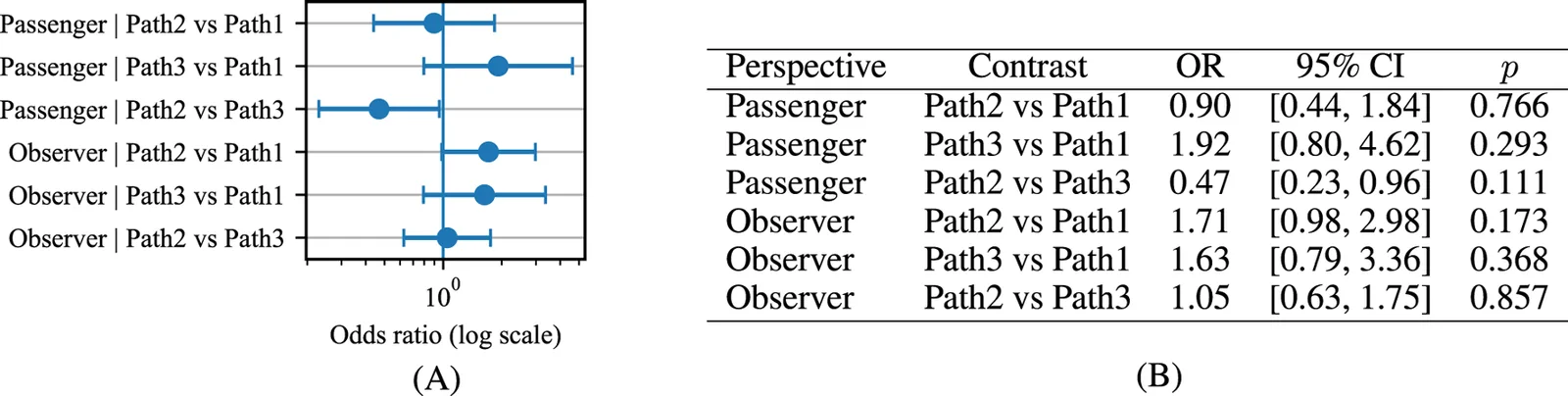
Environment effects within each perspective (EnvB vs. EnvA). The left panel presents the forest plot as odds ratios (log scale) with 95% confidence intervals based on participant-clustered sandwich standard errors. The right panel shows the corresponding *post hoc* statistics (odds ratios, 95% confidence intervals, and Holm-adjusted p values). **(A)** Forest plot. **(B)** Post hoc statistics.

In summary, no statistically significant environment effect was detected between the two tested corridor configurations.

### 5.5 Perspective-by-environment interaction effect (RQ4)

For RQ4, the omnibus test examined whether the perspective effect changed across environments. The omnibus null hypothesis was as given in Equation 9:

$$H_{0}:\;\Delta \Delta_{p}\left(\text{Path}2\text{ vs Path}1\right)=0\;\wedge \;\Delta \Delta_{p}\left(\text{Path}3\text{ vs Path}1\right)=0.$$ (9)

Descriptive results in Figure 4 suggest that the observer–passenger difference for the Path 2 vs. Path 3 contrast was larger in EnvA than in EnvB. The omnibus test did not support three-way interaction at the conventional 0.05 level, although the result was suggestive ( $\chi^{2}\left(2\right)=4.83$ , p=0.089 ; Table 1). Therefore, Figure 9 (panels A and B) presents descriptive interaction effect sizes rather than confirmed interaction effects. Among pairwise contrasts, the largest interaction estimate was for Path 2 vs. Path 3.

**FIGURE 9:**
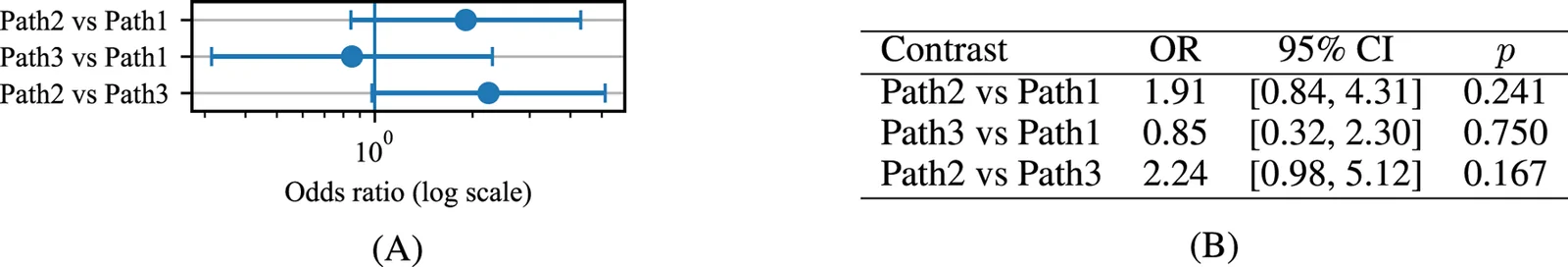
Three-way interaction expressed as a difference of differences ( $\Delta \Delta_{p}$ ; in-person experiment, n=35 ). The left panel presents the forest plot as odds ratios (log scale) with 95% confidence intervals based on participant-clustered sandwich standard errors. The right panel shows the corresponding *post hoc* statistics (odds ratios, 95% confidence intervals, and Holm-adjusted p values). **(A)** Forest plot. **(B)** Post hoc statistics.

In summary, the observer–passenger difference in path comfort did not vary significantly across corridor layouts, although the data suggest a possible trend. The larger interaction estimate for Path 2 vs. Path 3 is consistent with the findings in Sections 5.2 and 5.3 (where Path 2 vs. Path 3 differed only in EnvA–Passenger and the omnibus perspective effect was supported only in EnvA).

### 5.6 Descriptive analysis of optional free-text comments

Among the 35 participants, 26 provided at least one optional free-text comment. All available comments from these 26 participants were analyzed descriptively to contextualize the ranking results. The analysis used participant-level content coding: when a participant referred to the same category multiple times, that category was counted once for that participant. Ranking outcomes, such as whether a participant ranked Path 1, Path 2, or Path 3 first, were not used as coding categories. Instead, comments were first coded according to content, and the relation between the resulting content categories and the quantitative ranking pattern was examined after coding. Categories mentioned by at least five participants are reported in Table 3. Responses equivalent to “none” or “no particular comment” were not counted as no-or-weak-difference statements, because they could indicate either no perceived difference or that the relevant point had already been stated in another free-text response. Because the comments were optional, the analysis should be interpreted as exploratory contextualization rather than independent qualitative evidence.

**TABLE 3:** Content-based coding summary of optional free-text comments. Counts indicate the number of participants, out of the 26 who provided comments, whose comments included each category. Categories were not mutually exclusive, and each participant was counted once per category at most.

Category	Coding definition	n	Summary
Layout-change geometry and wall-continuity interpretation	Overall corridor layout and how its shape changed, including sudden versus gradual widening, diagonal or discontinuous wall geometry, and whether a path appeared congruent with the changing wall shape	19	Participants interpreted robot motion relative to the corridor’s geometric transition. Abrupt widening in Environment A sometimes made mid-course turns harder to predict, whereas gradual widening in Environment B made layout-congruent wall-following easier to understand or more natural. Several comments also indicated weak or absent environmental differences
Perspective-specific attention	Differences between passenger and observer viewpoints, including forward visibility, physical experience of heading change, corridor-wide visibility, wall proximity, and lateral placement	18	Passenger-perspective comments tended to emphasize local visual information, wall proximity, and the bodily experience of heading changes. Observer-perspective comments more often referred to lateral placement, corridor-wide geometry, and possible obstruction to other users
Motion simplicity and small path change	Straight motion, small path change, little lateral movement, few turns or rotations, little swaying, and absence of unnecessary movement	17	Most comments in this category favored straight, stable motion with little unnecessary movement. This category was broadly consistent with the high comfort rankings for Path 1, although a minority of comments valued visible path change when it made the robot’s intention easier to understand
Predictability, intention, and perceived control	Ease of predicting the robot’s movement, clarity of the robot’s intended path, and the impression that the robot recognized or responded to the environment	14	Participants often linked comfort to whether the path was understandable or predictable. Some associated predictability with straight motion, whereas others felt more comfortable when the path visibly responded to the changing corridor geometry
Local wall proximity and collision-related clearance	Local distance or clearance between the robot/path and nearby walls, perceived risk of contacting a wall, or visibility of nearby walls from the passenger view	13	Participants treated local wall clearance as a safety and comfort cue. Some comments linked close or poorly visible walls to anxiety or collision concern, whereas others described stable wall distance as reassuring. This category explains wall-distance-based individual differences, including some preferences for paths with greater wall clearance
Lateral movement, lateral placement, and centerline interpretation	Lateral movement, center-aligned motion, side-positioned motion, ambiguity regarding how to avoid the robot, and possible obstruction to other corridor users	9	Comments in this category described the comfort implications of centerline or side-positioned travel. Observer-perspective comments often treated center-aligned motion as obstructive or spatially ambiguous, although some participants felt reassured by center travel because it reduced wall-collision concerns
No or weak environmental difference stated	Comments explicitly stating that differences between Environment A and Environment B were weak, absent, or insufficient to change evaluations. Responses such as “none” or “no particular comment” were not included	9	Several participants explicitly reported weak or absent environmental differences, often for either the passenger or observer perspective. This category supports a cautious interpretation of the non-significant environment effect and prevents overinterpretation of layout-specific comments

The most frequent categories concerned layout-change geometry and wall-continuity interpretation (n=19) , perspective-specific attention (n=18) , and motion simplicity and small path change (n=17) . These categories provide exploratory context for the main quantitative patterns. The motion-simplicity category broadly agreed with the consistently high rankings observed for Path 1, which involved no heading change and maintained a simple side-positioned path. The perspective-specific category was consistent with the significant perspective effect in Environment A because passengers and observers appeared to focus on slightly different aspects of the same paths. The environment-geometry category indicated that some participants noticed layout-specific differences, but the no-or-weak-environment-difference category (n=9) also showed that several participants did not perceive a strong difference between the two corridor layouts. Therefore, the comment data contextualize the ranking results, but they should not be interpreted as confirmatory evidence for an environmental effect.

## 6 Discussion

### 6.1 Which tested path was most comfortable? (RQ1)

Path 1 was preferred most frequently in every Environment × Perspective condition, and this ordering was consistent across the inferential and descriptive analyses (Table 1; Figures 4, 6).

The descriptive comment analysis provides contextual support for this result. As shown in Table 3, many participants referred to motion simplicity, small path change, predictability, and wall distance as comfort criteria. These categories agree with the high ranking of Path 1, which combined no heading change, a stable lateral position, and a simple wall-following path.

This result is also relevant for path planners that default to center-aligned motion to maximize geometric clearance, such as obstacle-inflated cost maps or Voronoi-based planners (Lu et al., 2014; Garrido et al., 2006). In the examined varying-width corridors, the center-oriented Path 2 was not the most comfortable option in any condition. Thus, comfort-oriented planning in such environments may require criteria beyond geometric clearance alone, such as path simplicity, predictability, side positioning, and perceived collision risk.

### 6.2 Did path preferences differ between passenger and observer perspectives? (RQ2)

The analyses indicated a significant perspective effect in Environment A. Compared with passengers, observers showed a stronger preference for Path 3 over Path 1 and a weaker preference for Path 2 over Path 3.

The comment categories provide a plausible explanation for this perspective effect. Passenger-perspective comments tended to emphasize the physical experience of heading change, forward visibility, wall proximity, and unnecessary movement. These comments suggest that passengers may evaluate comfort partly through bodily experience and local visual information from the wheelchair. Under this interpretation, paths involving heading change or lateral movement can reduce passenger comfort even when they remain spatially reasonable from an external viewpoint.

Observer-perspective comments more frequently referred to lateral placement, corridor-wide geometry, and possible obstruction to other corridor users. In particular, several comments described center-aligned motion as potentially obstructive or spatially ambiguous. This pattern provides exploratory context for why observers rated Path 3 relatively higher than passengers did in Environment A: observers did not physically experience the heading change, and they could evaluate the robot’s side-positioned movement relative to the whole corridor. Note that the fixed observer position, at the corridor center after the width change, also linked center-aligned motion with a direct approach toward the observer, although few comments referred to the approach itself (Table 3).

This interpretation should be considered with caution because the comments were optional and descriptive. Nevertheless, the combined quantitative and comment results suggest that passenger comfort and observer comfort depend on partly different perceptual criteria. Within the tested setups, the findings suggest that reducing heading changes and unnecessary movement may be particularly important for passengers. For observers, heading change remains relevant, whereas avoiding prolonged center-aligned motion and maintaining an interpretable lateral position may serve as additional comfort cues.

### 6.3 Did path preferences differ between sudden and gradual width changes? (RQ3)

No statistically significant effect of corridor layout on path comfort was detected. Nevertheless, the optional free-text comments suggested a weak descriptive tendency: some participants found that abrupt widening in Environment A made turning paths harder to interpret, and others found that gradual widening in Environment B made wall-following easier to understand; several others reported little or no difference between environments. Because these comment-based impressions were not supported by the inferential analysis, they should be treated as descriptive rather than confirmatory. Moreover, because several participants stated that the difference between the two environments was weak or did not change their evaluations, the absence of a significant effect may reflect a weak environmental manipulation rather than an absence of influence of sudden versus gradual width changes in general, and the result should be regarded as specific to the two tested configurations (Section 7.3).

### 6.4 Did the perspective effect depend on corridor layout? (RQ4)

The Perspective × Environment interaction was not statistically significant. Thus, the present data do not support the conclusion that corridor layout reliably altered passenger–observer differences in path comfort. Nevertheless, as summarized in Table 3, the optional free-text comments included two relevant patterns: environment-related interpretability, such as abrupt widening in Environment A making mid-course turns harder to predict, and perspective-specific attention, such as observers focusing on lateral placement and possible obstruction to other users, whereas passengers focused on the bodily experience of heading change. These patterns are compatible with the larger descriptive observer–passenger difference in preferences between Path 2 and Path 3 in Environment A. However, because this overall pattern was not supported by the inferential analysis, it should be interpreted as descriptive rather than confirmatory.

### 6.5 Comparison with straight corridors of uniform width

The present findings are broadly consistent with earlier work on straight corridors of uniform width, such as Morales et al. (2013) and Robinson et al. (2024); however, they also refine that literature. Passenger-oriented studies in uniform corridors have demonstrated that the most comfortable lateral position is approximately 35% of the corridor width from the corridor edge rather than at the centerline (Morales et al., 2013). Observer-oriented work has also indicated that wall-following motion is evaluated more positively than non-wall-following motion (Robinson et al., 2024). In the present varying-width corridors, the center-oriented Path 2 was never the most comfortable option, and Path 1, which remained side-positioned, followed the wall, and avoided heading change, was consistently preferred (Figures 4, 6).

The contributions of this study go beyond a direct extension of the uniform-corridor literature to a different spatial configuration. Within the tested setups, the observed preference pattern is compatible with the possibility that heading change contributed more to perceived comfort than maintaining wall-following during the transition itself. This possibility is also consistent with the quantitative results in Environment B: although both Path 1 and Path 3 were wall-following paths, Path 1 was preferred as, in contrast to Path 3, it involved no heading change. Note that, because the tested paths were not designed to isolate these features, heading change and lateral position should be considered as possible factors contributing to the observed effects (Section 7.2).

### 6.6 Effects of pedestrian traffic rules

Robinson et al. (2024) reported that observer evaluations of wall-following in indoor corridors were not significantly affected by national driving-side conventions. Consistent with this finding, the present comment coding did not yield a recurring traffic-rule category, and only isolated comments referred to left-side traffic or culturally familiar side use. Nevertheless, because all participants were Japanese and the layouts were not mirrored, the present study cannot separate comfort based on path geometry from comfort based on side preference. Path 1 remained on the left after the corridor widened, whereas Path 2 and Path 3 shifted rightward. Future work should test mirrored layouts and cross-cultural samples.

### 6.7 Individual differences

Although Path 1 was preferred most frequently at the aggregate level, the comment analysis indicates meaningful individual differences in terms of comfort criteria. Comments regarding wall distance, lateral placement, predictability, and perceived control were particularly heterogeneous. Some participants preferred paths with greater wall clearance, which can explain cases where Path 2 received high rankings. Other participants preferred side-positioned wall-following paths because they appeared controlled, natural, or responsive to corridor geometry, which can explain cases where Path 3 received high rankings. Conversely, some participants evaluated Path 3 negatively when its heading change or lateral relocation appeared abrupt or hard to predict.

These individual differences suggest that Path 1 is a reasonable default choice for the tested scenarios but not a universally optimal path style. Long-term personal mobility systems may benefit from adaptive or user-specific path planning that considers whether a user prioritizes minimal movement, wall clearance, predictable side positioning, or apparent responsiveness to environmental geometry.

## 7 Limitations

### 7.1 Ranking method

The forced-ranking procedure elicited clear relative preferences among the three paths, but it may have amplified minor differences by requiring distinct ranks even when two paths were perceived as nearly equivalent. Additionally, ordinal rankings do not capture the magnitude of perceived differences. The current findings should therefore be interpreted as evidence of preference order rather than interval-scale differences in comfort.

### 7.2 Path factors were not fully disentangled

The candidate paths were selected to compare practically relevant path types in varying-width corridors rather than to isolate the causal contribution of individual geometric features. Nevertheless, the results suggest that several factors may influence comfort, including heading-change magnitude, lateral position, and wall-following behavior. Because these factors were combined within each path—for example, Path 1 combined minimal heading change with side positioning and wall-following, whereas Path 3 combined side positioning and wall-following with a larger turn—the present study cannot determine their independent contributions.

### 7.3 Strength of the environmental manipulation

The two environments were designed to contrast sudden and gradual width changes, but the differences actually experienced may have been subtle: nine participants explicitly stated that the difference between the environments was weak, absent, or insufficient to change their evaluations (Table 3). The non-significant environment effect and interaction therefore apply only to the two tested configurations, and future studies should use more strongly differentiated corridor geometries.

### 7.4 Participant sample

The participants were limited to young adults (aged 20–30 years), and the sample had a gender imbalance (28 males and 7 females). Moreover, all participants were Japanese students recruited from a single university. Because this work is an initial, exploratory study, this participant group was intended as a controlled initial sample for testing the proposed comparison rather than as a broadly representative population sample. Future work will focus on involving broader and more diverse participants across age groups, genders, and cultural backgrounds.

## 8 Conclusion

As an initial, exploratory study, this work provides context-specific empirical evidence for developing path-design guidelines to improve comfort during navigation in varying-width corridors from both passenger and observer perspectives. An in-person experiment (n=35) compared three paths (a straight wall-following path, a center path with a small heading change, and a wall-following path with a larger heading change) across two perspectives (passenger, observer) and two layouts (sudden, gradual width change), with comfort measured as complete path rankings and analyzed using a PL model. Across all conditions, the straight wall-following path was most preferred. In the sudden-change layout, preferences differed significantly according to perspective: passengers preferred the center path over the path with the larger heading change, whereas observers showed no such preference and rated the latter relatively higher. No significant effect of layout (sudden vs. gradual width change) or perspective-by-layout interaction was detected. Taken together, these findings suggest two path-design implications for the tested scenarios: paths with minimal heading change can provide a useful default, and less center-aligned motion may merit consideration when observer comfort is important.

In addition to addressing the limitations discussed in Section 7, future work should extend these findings in two directions. First, studies should vary observer position to test how viewpoint-dependent visual information affects observer comfort perceptions. Second, studies should model individual differences, such as personality traits, to clarify how user attributes relate to comfort preferences and to support personalized path planning.

## Data Availability

The datasets presented in this article are not readily available because ongoing related follow-up studies. Requests to access the datasets should be directed to the corresponding author.
