# Supplementary material for: Evaluating the comfort of robot paths in varying-width corridors: passenger vs. observer perspectives
Source: Front Robot AI. 2026 Oct 29;13:1971439. doi: 10.3389/frobt.2026.1971439 (PMC13755837; doi:10.3389/frobt.2026.1971439)
Supplement: Supplementary file 1 [file DataSheet1.pdf]

Supplementary Material

1 SUPPLEMENTARY TABLES AND FIGURES

1.1 Figures

Figures S1 and S2 show photographs of the experimental scenes for Environments A and B, respectively, from the passenger and observer perspectives for each of the three paths. The corridor layouts, the three paths, and the observer position are illustrated in Figure 3 of the main text.

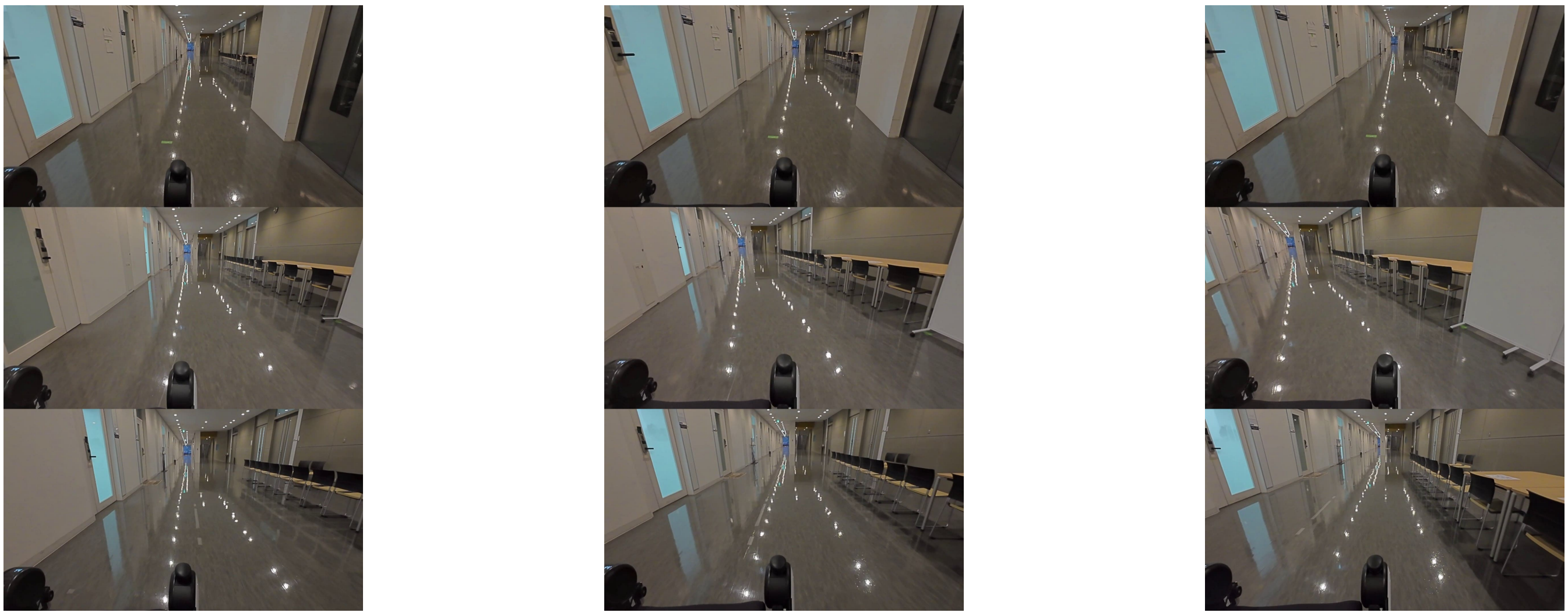

(i) Path 1 (ii) Path 2 (iii) Path 3

(A) Passenger perspective

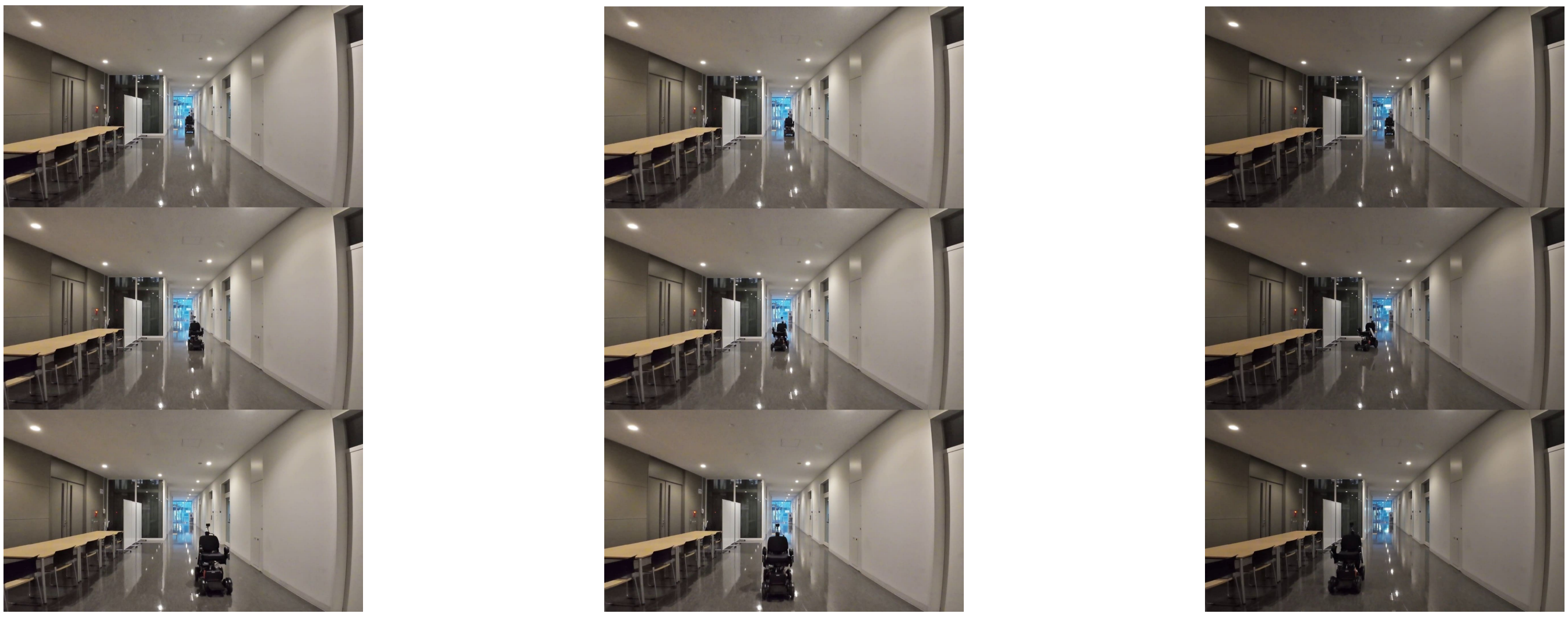

(i) Path 1 (ii) Path 2 (iii) Path 3

(B) Observer perspective

Figure S1. Experimental scene in Environment A: photographs of the passenger and observer views for the three paths (the layout is shown in Figure 3 of the main text). The robot navigates through a corridor with a sudden change in width, inducing an abrupt shift in the passenger's perceived spatial layout. This may cause the passenger's viewpoint to change dynamically as the robot moves.

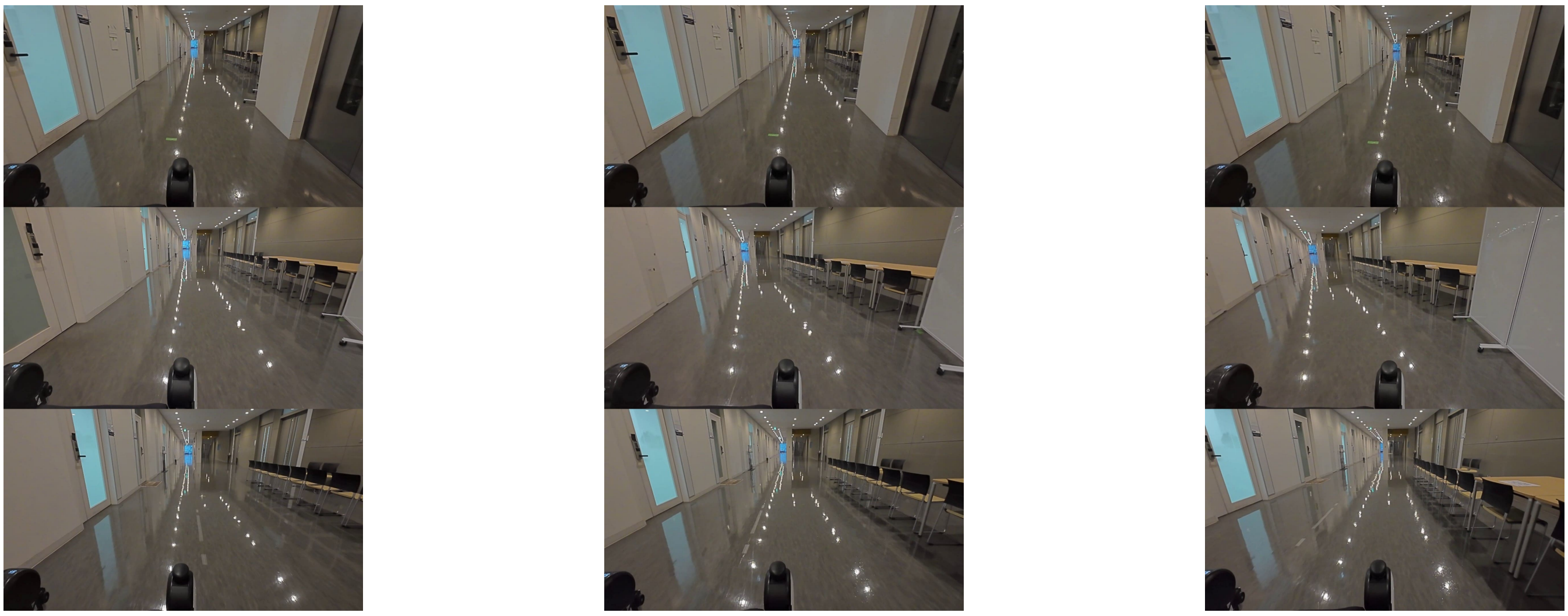

(i) Path 1 (ii) Path 2 (iii) Path 3

(A) Passenger perspective

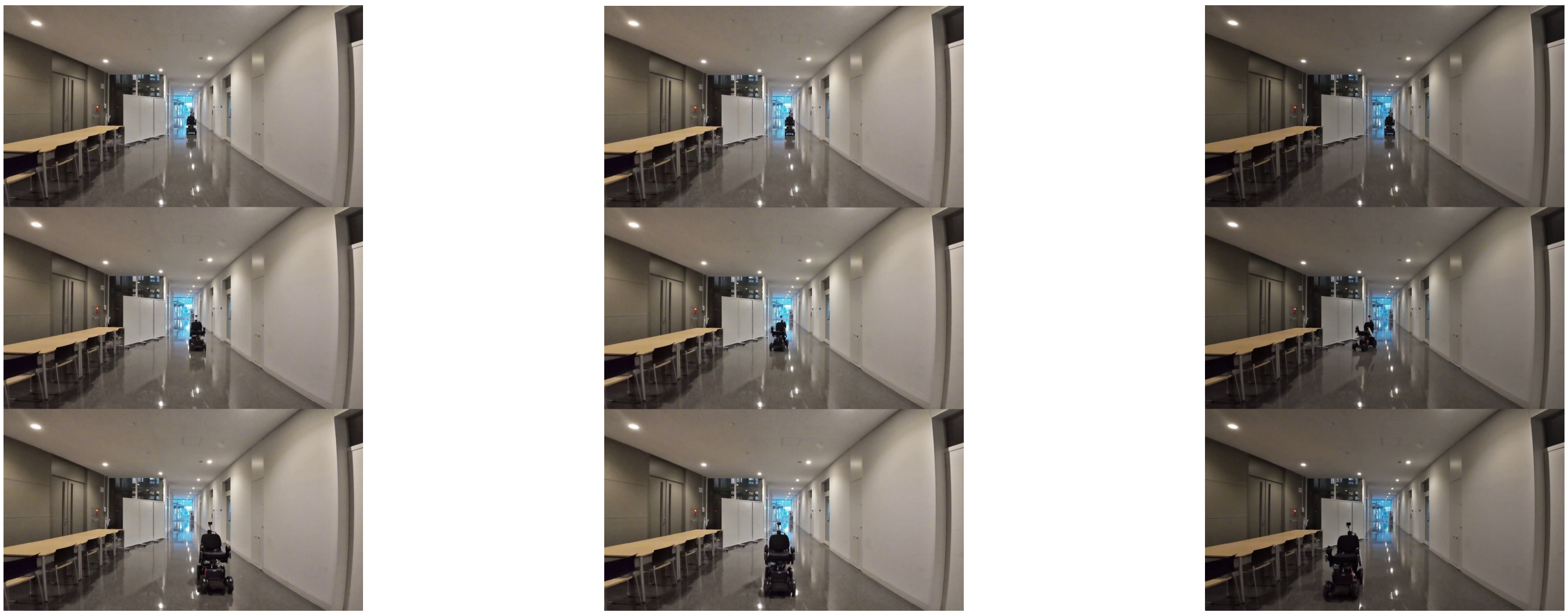

(i) Path 1 (ii) Path 2 (iii) Path 3

(B) Observer perspective

Figure S2. Experimental scene in Environment B: photographs of the passenger and observer views for the three paths (the layout is shown in Figure 3 of the main text). The corridor width changes gradually, providing a more continuous but still dynamic shift in the perceived spatial layout from the passenger's viewpoint as the robot moves forward.

1.2 Tables

Table S1 lists the videos of the passenger and observer views for all environment–path combinations used in the experiment.

Table S1. Videos of the passenger and observer views for each environment and path.

Environment	Perspective	Path	URL
Environment A	Passenger	Path 1	`https://youtu.be/efBfnP5Zgu8`
Environment A	Passenger	Path 2	`https://youtu.be/8YTd1hqH9vg`
Environment A	Passenger	Path 3	`https://youtu.be/IKsMKSWYngA`
Environment A	Observer	Path 1	`https://youtu.be/1psUxMtDo_E`
Environment A	Observer	Path 2	`https://youtu.be/wMv1MzHPxJA`
Environment A	Observer	Path 3	`https://youtu.be/omYdSAelkkE`
Environment B	Passenger	Path 1	`https://youtu.be/z-TTOcdGexQ`
Environment B	Passenger	Path 2	`https://youtu.be/O6iC3O77Fr4`
Environment B	Passenger	Path 3	`https://youtu.be/LbFfLWUmm8c`
Environment B	Observer	Path 1	`https://youtu.be/VuB5Gl1h1nk`
Environment B	Observer	Path 2	`https://youtu.be/nOXAUz4P_d0`
Environment B	Observer	Path 3	`https://youtu.be/dDqodzLhC9s`
